# Supplementary material for: Loss of Kmt2c or Kmt2d drives brain metastasis via KDM6A-dependent upregulation of MMP3
Source: Nat Cell Biol. 2024 Jun 26;26(7):1165–75. doi: 10.1038/s41556-024-01446-3 (PMC11251985; doi:10.1038/s41556-024-01446-3)
Supplement: Supplementary file 1 — Supplementary Fig, 1. [file 41556_2024_1446_MOESM1_ESM.pdf]

nature cell biology

Supplementary information

https://doi.org/10.1038/s41556-024-01446-3

Loss of *Kmt2c* or *Kmt2d* drives brain metastasis via KDM6A-dependent upregulation of MMP3

In the format provided by the authors and unedited

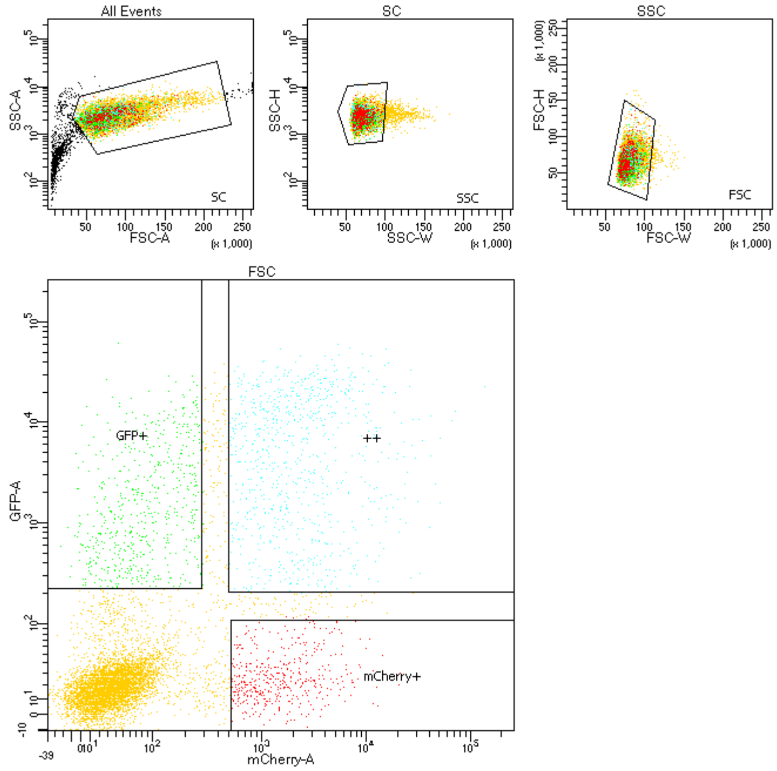

Tube: 2

Population	#Events	%Parent	%Total
All Events	10,000	####	100.0
SC	8,942	89.4	89.4
SSC	8,407	94.0	84.1
FSC	8,283	98.5	82.8
++	818	9.9	8.2
GFP+	594	7.2	5.9
mCherry+	450	5.4	4.5

gating strategy for sorting GFP/mCherry double positive cells after transfection with sgRNA-mCherry and Cas9-GFP plasmids described in Fig 1b

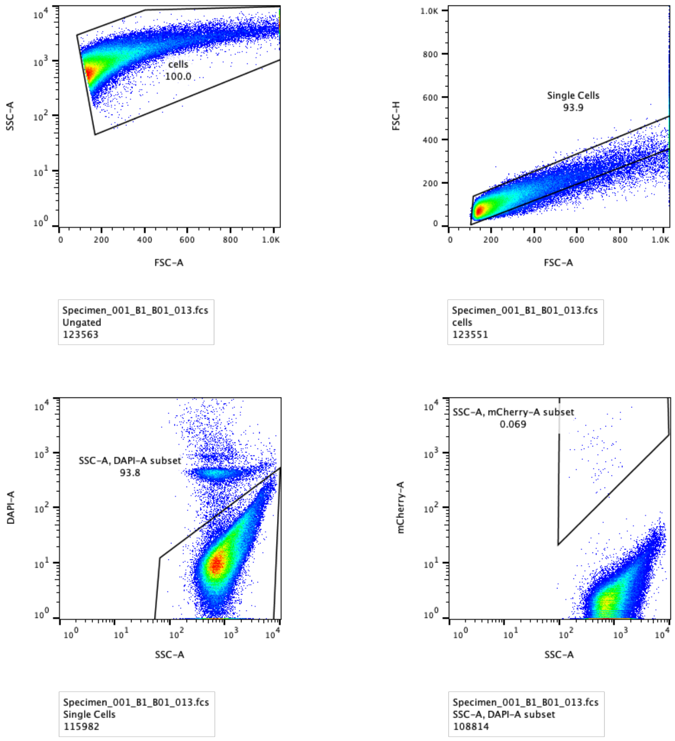

gating strategy for identifying mCherry positive/ DAPI negative metastases from digested whole organ cell solutions as depicted in Extended Data Fig 1n

Supplementary Figure 1
