# Supplementary material for: Loss of Kmt2c or Kmt2d drives brain metastasis via KDM6A-dependent upregulation of MMP3
Source: Nat Cell Biol. 2024 Jun 26;26(7):1165–75. doi: 10.1038/s41556-024-01446-3 (PMC11251985; doi:10.1038/s41556-024-01446-3)
Supplement: Supplementary file 2 — Reporting Summary [file 41556_2024_1446_MOESM2_ESM.pdf]

nature portfolio

Corresponding author(s): Kornelia Polyak

Last updated by author(s): 05/06/2024

Reporting Summary

Nature Portfolio wishes to improve the reproducibility of the work that we publish. This form provides structure for consistency and transparency in reporting. For further information on Nature Portfolio policies, see our Editorial Policies and the Editorial Policy Checklist.

Statistics

For all statistical analyses, confirm that the following items are present in the figure legend, table legend, main text, or Methods section.

n/a	Confirmed	
☐	☒	The exact sample size (*n*) for each experimental group/condition, given as a discrete number and unit of measurement
☐	☒	A statement on whether measurements were taken from distinct samples or whether the same sample was measured repeatedly
☐	☒	The statistical test(s) used AND whether they are one- or two-sided<br>*Only common tests should be described solely by name; describe more complex techniques in the Methods section.*
☒	☐	A description of all covariates tested
☐	☒	A description of any assumptions or corrections, such as tests of normality and adjustment for multiple comparisons
☐	☒	A full description of the statistical parameters including central tendency (e.g. means) or other basic estimates (e.g. regression coefficient) AND variation (e.g. standard deviation) or associated estimates of uncertainty (e.g. confidence intervals)
☐	☒	For null hypothesis testing, the test statistic (e.g. *F*, *t*, *r*) with confidence intervals, effect sizes, degrees of freedom and *P* value noted<br>*Give P values as exact values whenever suitable.*
☒	☐	For Bayesian analysis, information on the choice of priors and Markov chain Monte Carlo settings
☒	☐	For hierarchical and complex designs, identification of the appropriate level for tests and full reporting of outcomes
☒	☐	Estimates of effect sizes (e.g. Cohen's *d*, Pearson's *r*), indicating how they were calculated

Our web collection on statistics for biologists contains articles on many of the points above.

Software and code

Policy information about availability of computer code

Data collection	scRNAseq: About 26,000 single cells were loaded onto a 10x Genomics ChromiumTM instrument (10x Genomics) according to the manufacturer's recommendations. The scRNAseq libraries were generated using Chromium Next GEM Single Cell 5' HT Kit v2 (10x Genomics). Demultiplexing, barcoded processing, feature counting and aggregation was done using cellranger v7.0.1.<br>ChIPseq: ChIPseq libraries were prepared using xGen DNA library prep reagents (IDT) on a Biomek i7 (Beckman Coulter) liquid handling platform from approximately 1ng of DNA with 14 cycles of PCR amplification according to manufacturer's protocol. Fastq data was processed using CoBRA pipeline (cobra 2.0, https://bitbucket.org/cfce/cobra/src/master/).<br>Steps include alignment using BWA mem, filtering for uniquely mapped read and sorting bam files. Peaks were called using macs2 with default settings (-broad mode for H3K4me1 and H3K27me3). Output bedgraph files were then used to generate bigWig files.<br>RNAseq: libraries were prepared from 100 ng RNA using Kapa mRNA HyperPrep (Roche) according to manufacturers protocol. VIPER pipeline (https://github.com/hanfeisun/viper-rnaseq/blob/master/cfce/README_CFCE.md) was used for alignment, assembly and gene counting.<br>Flow cytometry: Single cells were acquired using a 4-laser and 17-parameter BD LSR Fortessa Cell Analyzer
Data analysis	scRNAseq: About 26,000 single cells were loaded onto a 10x Genomics ChromiumTM instrument (10x Genomics) according to the manufacturer's recommendations. The scRNAseq libraries were generated using Chromium gNext GEM Single Cell 5' HT Kit v2 (10x Genomics). Demultiplexing, barcoded processing, feature counting and aggregation was done using cellranger v7.0.1.<br>ChIPseq: ChIPseq libraries were prepared using xGen DNA library prep reagents (IDT) on a Biomek i7 (Beckman Coulter) liquid handling platform from approximately 1ng of DNA with 14 cycles of PCR amplification according to manufacturer's protocol. Fastq data was processed using CoBRA pipeline (https://bitbucket.org/cfce/cobra/src/master/).<br>Steps include alignment using BWA mem, filtering for uniquely mapped read and sorting bam files. Peaks were called using macs2 with default settings (-broad mode for H3K4me1 and H3K27me3). Output bedgraph files were then used to generate bigWig files.<br>RNAseq: libraries were prepared from 100 ng RNA using Kapa mRNA HyperPrep (Roche) according to manufacturers protocol. VIPER pipeline

(https://github.com/hanfeisun/viper-rnaseq/blob/master/cfce/README_CFCE.md) was used for alignment, assembly and gene counting.

R studio 2022.10.0-daily+67, seurat 4.3.0, DEseq2 1.38.3, GSEA 4.2.1, Graph Pad Prism 10, FlowJo 10.9.0, Fiji v1.54f

For manuscripts utilizing custom algorithms or software that are central to the research but not yet described in published literature, software must be made available to editors and reviewers. We strongly encourage code deposition in a community repository (e.g. GitHub). See the Nature Portfolio guidelines for submitting code & software for further information.

Data

Policy information about availability of data

All manuscripts must include a data availability statement. This statement should provide the following information, where applicable:

- Accession codes, unique identifiers, or web links for publicly available datasets
- A description of any restrictions on data availability
- For clinical datasets or third party data, please ensure that the statement adheres to our policy

All data needed to evaluate the conclusions in the paper are present in the paper and/or the Supplemental Information. . All raw and processed genomic data was deposited to GEO under accession number: GSE237392.
Mass spectrometry data have been deposited in ProteomeXchange with the primary accession code PXD052075 https://proteomecentral.proteomexchange.org/cgi/GetDataset?ID=PXD052075.

Research involving human participants, their data, or biological material

Policy information about studies with human participants or human data. See also policy information about sex, gender (identity/presentation), and sexual orientation and race, ethnicity and racism.

Reporting on sex and gender	N/A
Reporting on race, ethnicity, or other socially relevant groupings	N/A
Population characteristics	N/A
Recruitment	N/A
Ethics oversight	N/A

Note that full information on the approval of the study protocol must also be provided in the manuscript.

Field-specific reporting

Please select the one below that is the best fit for your research. If you are not sure, read the appropriate sections before making your selection.

☒ Life sciences ☐ Behavioural & social sciences ☐ Ecological, evolutionary & environmental sciences

For a reference copy of the document with all sections, see nature.com/documents/nr-reporting-summary-flat.pdf

Life sciences study design

All studies must disclose on these points even when the disclosure is negative.

Sample size	Sample sizes for mouse experiments, cell culture and sequencing experiments were chosen according to former experiences with similar experiments to reach sufficient statistical power (see Shu et al. "Molecular cell (2020) or Janiszewska et al. Nature cell biology (2019)).
Data exclusions	For mouse metastases models, mice with failed intracardiac injection were excluded. Failed injection (wrong ventricle injection) was specified as the onset of high metastatic burden exclusively in the lung but no other tissue 4 - 5 days before collection of the remaining comparable group. For ChIP and scRNAseq low quality reads were excluded as described in the method and quality control section.
Replication	All in vitro and sequencing experiments were conducted with biological replicates (defined as 1 passaging (~5 days) between collection of cell line replicates). Experiments were replicated 2-4 times as indicated in the according figure legend. All replications were included in the data analysis. Mouse experiments were not individually replicated to comply with the 3R principle for animal research, however, sufficient samples sizes were chosen for each experiment.
Randomization	For in vivo studies mice were randomly allocated to injection of different cell lines. No other randomization was used.
Blinding	Sample collection for mouse experiments was not blinded, however, samples were randomized before flow cytometry and imaging and allocated to samples afterwards. Other experiments were not blinded as it would not affect results or interpretation.

Reporting for specific materials, systems and methods

We require information from authors about some types of materials, experimental systems and methods used in many studies. Here, indicate whether each material, system or method listed is relevant to your study. If you are not sure if a list item applies to your research, read the appropriate section before selecting a response.

Materials & experimental systems

n/a	Involved in the study
☐	☒ Antibodies
☐	☒ Eukaryotic cell lines
☒	☐ Palaeontology and archaeology
☐	☒ Animals and other organisms
☒	☐ Clinical data
☒	☐ Dual use research of concern
☒	☐ Plants

Methods

n/a	Involved in the study
☐	☒ ChIP-seq
☐	☒ Flow cytometry
☒	☐ MRI-based neuroimaging

Antibodies

Antibodies used

KMT2C rb 1/1000 gift from Ali Shilatifard western blot
KMT2D rb 1/1000 Biorbyt orb184541 western blot
tubulin ms 1/20000 Millipore Sigma T6199 western blot
total histone H3 ms 1/1000 Active motif 39763 western blot
H3K4me1 rb 1/1000 Invitrogen 710795 western blot
H3K27me3 rb 1/1000 Active motif 39155 western blot
H3K27ac rb 1/1000 abcam ab4729 western blot
KDM6A rb 1/1000 Cell Signaling 33510S western blot
PAXIP1 rb 1/1000 Sigma Aldrich ABE1877 western blot
RBBP5 rb 1/1000 Cell Signaling 13171S western blot
WDR5 rb 1/1000 Cell Signaling 13105S western blot
KDM6A rb 2.5 ug/IP Cell Signaling 33510S immunoprecipitation
H3K4me1 rb 2.5 ug/ChIP abcam ab8895 ChIP
H3K27me3 rb 2.5 ug/ChIP Cell Signaling 9733S ChIP
H3K27ac rb 2.5 ug/ChIP Diagenode C15410196 ChIP
KDM6A rb 2.5 ug/ChIP Cell Signaling 33510S ChIP
P300 rb 2.5 ug/ChIP abcam ab275378 ChIP
CD8 rb 1/500 Cell Signaling 98941S immunofluorescence
PDL-1 rb 1/200 Cell Signaling 64988S immunofluorescence
mCherry rb 1/500 Cell Signaling 43590S immunofluorescence
rb anti goat-HRP 1/10000 Invitrogen 65-6120 western blot
ms anti goat-HRP 1/10000 Invitrogen 62-6520 western blot
rb anti goat Alexa Fluor 6471/500 Invitrogen A-21245 immunofluorescence
rb anti goat Alexa Fluor 555 1/500 Invitrogen A-21428 immunofluorescence

Validation

Validation of all the commercial antibodies can be found on the manufacturer's website using the provided catalog number.
Antibodies for ChIPseq were validated using the analysis QC control.

Specific validation of antibodies:
KMT2C/KMT2D orb184541 - validated with confirmed knockout samples in this manuscript
tubulin T6199 several enhanced validation approaches https://www.sigmaaldrich.com/US/en/product/sigma/t6199
total histone H3 39763 This antibody has been validated for use in ChIP and/or ChIP-Seq https://www.activemotif.com/catalog/details/39763
H3K4me1 710795 This Antibody was verified by Peptide array to ensure that the antibody binds to the antigen stated. https://www.thermofisher.com/antibody/product/H3K4me1-Antibody-Recombinant-Polyclonal/710795
H3K27me3 39155 This antibody has been validated for use in ChIP and/or ChIP-Seq https://www.activemotif.com/catalog/details/39155
H3K27ac ab4729 Suitable for: ICC/IF, WB, IHC-P, ChIP, PepArr https://www.abcam.com/products/primary-antibodies/histone-h3-acetyl-k27-antibody-chip-grade-ab4729.html
KDM6A 33510S validation via western blot of known positive and negative samples https://www.cellsignal.com/products/primary-antibodies/utx-d3q1i-rabbit-mab/33510
PAXIP1 ABE1877 Evaluated by Western Blotting with with recombinant Pax-interacting protein 1. https://www.emdmillipore.com/US/en/product/Anti-PAXIP1,MM_NF-ABE1877
RBBP5 13171S validation via immunoprecipitation https://www.cellsignal.com/products/primary-antibodies/rbbp5-d3i6p-rabbit-mab/13171
WDR5 13105S This antibody has been validated using SimpleChIP® Enzymatic Chromatin IP Kits. https://www.cellsignal.com/products/primary-antibodies/wdr5-d9e1i-rabbit-mab/13105
KDM6A 33510S validation via western blot of known positive and negative samples https://www.cellsignal.com/products/primary-antibodies/utx-d3q1i-rabbit-mab/33510
H3K4me1 ab8895 confirmed specificity through extensive validation https://www.abcam.com/products/primary-antibodies/histone-

h3-mono-methyl-k4-antibody-chip-grade-ab8895.html
H3K27me3 9733S tested for specificity and cross reactions https://www.cellsignal.com/products/primary-antibodies/tri-methyl-histone-h3-lys27-c36b11-rabbit-mab/9733
H3K27ac C15410196 validation via immunoprecipitation, ChIP and cross-ractivity studies https://www.diagenode.com/en/p/h3k27ac-polyclonal-antibody-premium-50-mg-18-ml
KDM6A 33510S validation via western blot of known positive and negative samples https://www.cellsignal.com/products/primary-antibodies/utx-d3q1i-rabbit-mab/33510
P300 ab275378 validation via immunoprecipitation https://www.abcam.com/products/primary-antibodies/kat3b--p300-antibody-epr23495-268-chip-grade-ab275378.html
CD8 98941S validation via western blot of known positive and negative samples https://www.cellsignal.com/products/primary-antibodies/cd8a-d4w2z-xp-rabbit-mab/98941
PDL-1 64988S specificity has been validated in known samples in this study https://www.cellsignal.com/products/primary-antibodies/pd-l1-d5v3b-rabbit-mab/64988
mCherry 43590S validation via western blot of known positive and negative samples https://www.cellsignal.com/products/primary-antibodies/mcherry-e5d8f-rabbit-mab/43590
rb 65-6120 tested for specificity and cross reactions https://www.thermofisher.com/antibody/product/Goat-anti-Rabbit-IgG-H-L-Secondary-Antibody-Polyclonal/65-6120
ms 62-6520 tested for specificity and cross reactions https://www.thermofisher.com/antibody/product/Goat-anti-Mouse-IgG-H-L-Secondary-Antibody-Polyclonal/62-6520
rb A-21245 whole antibodies have been cross-adsorbed https://www.thermofisher.com/antibody/product/Goat-anti-Rabbit-IgG-H-L-Highly-Cross-Adsorbed-Secondary-Antibody-Polyclonal/A-21245
rb A-21428 whole antibodies have been cross-adsorbed https://www.thermofisher.com/antibody/product/Goat-anti-Rabbit-IgG-H-L-Cross-Adsorbed-Secondary-Antibody-Polyclonal/A-21428

Eukaryotic cell lines

Policy information about cell lines and Sex and Gender in Research

Cell line source(s)
168FARN and 67NR murine mammary tumor cell lines were obtained from the Karmanos Cancer Institute, HEK293T cells were obtained from ATCC and cultured following the provider's recommendations

Authentication
168FARN and 67NR murine mammary tumor cell lines were directly obtained from the Karmanos Cancer Institute, HEK293T cells were directly obtained from ATCC. Thus, no further authentication has been done.

Mycoplasma contamination
Cell were frequently tested negative for mycoplasma using PCR-based assays.

Commonly misidentified lines (See ICLAC register)
No commonly misidentified cell line was used.

Animals and other research organisms

Policy information about studies involving animals; ARRIVE guidelines recommended for reporting animal research, and Sex and Gender in Research

Laboratory animals
For mammary fatpad injection and intracardiac seeding female BALB/c or NOD.Cg-Prkdcscid Il2rgtm1Wjl/SzJ (NSG) mice were purchased from The Jackson Laboratory at 5-6 weeks of age.

Wild animals
The study did not involve wild animals.

Reporting on sex
All experiments were done in female mice only. Breast cancer rarely occurs in males thus research with female animals can be justified.

Field-collected samples
The study did not involve field-collected samples.

Ethics oversight
Dana-Farber Cancer Institute IACUC

Note that full information on the approval of the study protocol must also be provided in the manuscript.

Plants

Seed stocks
Report on the source of all seed stocks or other plant material used. If applicable, state the seed stock centre and catalogue number. If plant specimens were collected from the field, describe the collection location, date and sampling procedures.

Novel plant genotypes
Describe the methods by which all novel plant genotypes were produced. This includes those generated by transgenic approaches, gene editing, chemical/radiation-based mutagenesis and hybridization. For transgenic lines, describe the transformation method, the number of independent lines analyzed and the generation upon which experiments were performed. For gene-edited lines, describe the editor used, the endogenous sequence targeted for editing, the targeting guide RNA sequence (if applicable) and how the editor was applied.

Authentication
Describe any authentication procedures for each seed stock used or novel genotype generated. Describe any experiments used to assess the effect of a mutation and, where applicable, how potential secondary effects (e.g. second site T-DNA insertions, mosiacism, off-target gene editing) were examined.

ChIP-seq

Data deposition

☒ Confirm that both raw and final processed data have been deposited in a public database such as GEO.

☒ Confirm that you have deposited or provided access to graph files (e.g. BED files) for the called peaks.

Data access links
May remain private before publication.

The following secure token has been created to allow review of record GSE237392 while it remains in private status: snwbeyicbtqffot

Files in database submission

20221207_MS11_MS10569_S163_R1_001.fastq.gz
20221207_MS11_MS10569_S163_R2_001.fastq.gz
20221207_MS20_MS10569_S29_R1_001.fastq.gz
20221207_MS20_MS10569_S29_R2_001.fastq.gz
20221207_MS21_MS10569_S30_R1_001.fastq.gz
20221207_MS21_MS10569_S30_R2_001.fastq.gz
20221207_MS26_MS10569_S34_R1_001.fastq.gz
20221207_MS26_MS10569_S34_R2_001.fastq.gz
20221207_MS39_MS10569_S65_R1_001.fastq.gz
20221207_MS39_MS10569_S65_R2_001.fastq.gz
20221207_MS40_MS10569_S66_R1_001.fastq.gz
20221207_MS40_MS10569_S66_R2_001.fastq.gz
20221207_MS51_MS10569_S76_R1_001.fastq.gz
20221207_MS51_MS10569_S76_R2_001.fastq.gz
20221207_MS52_MS10569_S77_R1_001.fastq.gz
20221207_MS52_MS10569_S77_R2_001.fastq.gz
20221207_MS53_MS10569_S78_R1_001.fastq.gz
20221207_MS53_MS10569_S78_R2_001.fastq.gz
20221207_MS54_MS10569_S79_R1_001.fastq.gz
20221207_MS54_MS10569_S79_R2_001.fastq.gz
20221207_MS55_MS10569_S80_R1_001.fastq.gz
20221207_MS55_MS10569_S80_R2_001.fastq.gz
20221208_MS50_MS10569R_S43_R1_001.fastq.gz
20221208_MS50_MS10569R_S43_R2_001.fastq.gz
220517_MS1_ACAGTG_MS10024_S5_R1_001.fastq.gz
220517_MS1_ACAGTG_MS10024_S5_R2_001.fastq.gz
220517_MS1_ACTTGA_MS10024_S8_R1_001.fastq.gz
220517_MS1_ACTTGA_MS10024_S8_R2_001.fastq.gz
220517_MS1_AGTCAA_MS10024_S13_R1_001.fastq.gz
220517_MS1_AGTCAA_MS10024_S13_R2_001.fastq.gz
220517_MS1_AGTTCC_MS10024_S14_R1_001.fastq.gz
220517_MS1_AGTTCC_MS10024_S14_R2_001.fastq.gz
220517_MS1_ATCACG_MS10024_S1_R1_001.fastq.gz
220517_MS1_ATCACG_MS10024_S1_R2_001.fastq.gz
220517_MS1_ATGTCA_MS10024_S15_R1_001.fastq.gz
220517_MS1_ATGTCA_MS10024_S15_R2_001.fastq.gz
220517_MS1_CAGATC_MS10024_S7_R1_001.fastq.gz
220517_MS1_CAGATC_MS10024_S7_R2_001.fastq.gz
220517_MS1_CCGTCC_MS10024_S16_R1_001.fastq.gz
220517_MS1_CCGTCC_MS10024_S16_R2_001.fastq.gz
220517_MS1_CGATGT_MS10024_S2_R1_001.fastq.gz
220517_MS1_CGATGT_MS10024_S2_R2_001.fastq.gz
220517_MS1_CTTGTA_MS10024_S12_R1_001.fastq.gz
220517_MS1_CTTGTA_MS10024_S12_R2_001.fastq.gz
220517_MS1_GATCAG_MS10024_S9_R1_001.fastq.gz
220517_MS1_GATCAG_MS10024_S9_R2_001.fastq.gz
220517_MS1_GCCAAT_MS10024_S6_R1_001.fastq.gz
220517_MS1_GCCAAT_MS10024_S6_R2_001.fastq.gz
220517_MS1_GGCTAC_MS10024_S11_R1_001.fastq.gz
220517_MS1_GGCTAC_MS10024_S11_R2_001.fastq.gz
220517_MS1_GTCCGC_MS10024_S17_R1_001.fastq.gz
220517_MS1_GTCCGC_MS10024_S17_R2_001.fastq.gz
220517_MS1_GTGAAA_MS10024_S18_R1_001.fastq.gz
220517_MS1_GTGAAA_MS10024_S18_R2_001.fastq.gz
220517_MS1_TAGCTT_MS10024_S10_R1_001.fastq.gz
220517_MS1_TAGCTT_MS10024_S10_R2_001.fastq.gz
220517_MS1_TGACCA_MS10024_S4_R1_001.fastq.gz
220517_MS1_TGACCA_MS10024_S4_R2_001.fastq.gz
220517_MS1_TTAGGC_MS10024_S3_R1_001.fastq.gz
220517_MS1_TTAGGC_MS10024_S3_R2_001.fastq.gz
220517_MS2_ACTTGA_MS10024_S26_R1_001.fastq.gz
220517_MS2_ACTTGA_MS10024_S26_R2_001.fastq.gz
220517_MS2_AGTCAA_MS10024_S31_R1_001.fastq.gz

220517_MS2_AGTCAA_MS10024_S31_R2_001.fastq.gz
220517_MS2_AGTTCC_MS10024_S32_R1_001.fastq.gz
220517_MS2_AGTTCC_MS10024_S32_R2_001.fastq.gz
220517_MS2_ATCACG_MS10024_S19_R1_001.fastq.gz
220517_MS2_ATCACG_MS10024_S19_R2_001.fastq.gz
220517_MS2_ATGTCA_MS10024_S33_R1_001.fastq.gz
220517_MS2_ATGTCA_MS10024_S33_R2_001.fastq.gz
220517_MS2_CCGTCC_MS10024_S34_R1_001.fastq.gz
220517_MS2_CCGTCC_MS10024_S34_R2_001.fastq.gz
220517_MS2_CTTGTA_MS10024_S30_R1_001.fastq.gz
220517_MS2_CTTGTA_MS10024_S30_R2_001.fastq.gz
220517_MS2_GATCAG_MS10024_S27_R1_001.fastq.gz
220517_MS2_GATCAG_MS10024_S27_R2_001.fastq.gz
220517_MS2_GCCAAT_MS10024_S24_R1_001.fastq.gz
220517_MS2_GCCAAT_MS10024_S24_R2_001.fastq.gz
220517_MS2_GGCTAC_MS10024_S29_R1_001.fastq.gz
220517_MS2_GGCTAC_MS10024_S29_R2_001.fastq.gz
220517_MS2_GTCCGC_MS10024_S35_R1_001.fastq.gz
220517_MS2_GTCCGC_MS10024_S35_R2_001.fastq.gz
220517_MS2_GTGAAA_MS10024_S36_R1_001.fastq.gz
220517_MS2_GTGAAA_MS10024_S36_R2_001.fastq.gz
220517_MS2_TAGCTT_MS10024_S28_R1_001.fastq.gz
220517_MS2_TAGCTT_MS10024_S28_R2_001.fastq.gz
220517_MS2_TGACCA_MS10024_S22_R1_001.fastq.gz
220517_MS2_TGACCA_MS10024_S22_R2_001.fastq.gz
220517_MS2_TTAGGC_MS10024_S21_R1_001.fastq.gz
220517_MS2_TTAGGC_MS10024_S21_R2_001.fastq.gz
122623_MSe01_MS11732_S33_R1_001.fastq.gz
122623_MSe01_MS11732_S33_R2_001.fastq.gz
122623_MSe02_MS11732_S34_R1_001.fastq.gz
122623_MSe02_MS11732_S34_R2_001.fastq.gz
122623_MSe03_MS11732_S35_R1_001.fastq.gz
122623_MSe03_MS11732_S35_R2_001.fastq.gz
122623_MSe06_MS11732_S38_R1_001.fastq.gz
122623_MSe06_MS11732_S38_R2_001.fastq.gz
122623_MSe07_MS11732_S39_R1_001.fastq.gz
122623_MSe07_MS11732_S39_R2_001.fastq.gz
122623_MSe08_MS11732_S40_R1_001.fastq.gz
122623_MSe08_MS11732_S40_R2_001.fastq.gz
122623_MSe10_MS11732_S42_R1_001.fastq.gz
122623_MSe10_MS11732_S42_R2_001.fastq.gz
122623_MSe11_MS11732_S43_R1_001.fastq.gz
122623_MSe11_MS11732_S43_R2_001.fastq.gz
122623_MSe12_MS11732_S44_R1_001.fastq.gz
122623_MSe12_MS11732_S44_R2_001.fastq.gz
220517_MS1_AGTCAA_MS10024_S13_R1_001.bw
220517_MS1_AGTTCC_MS10024_S14_R1_001.bw
220517_MS1_ATGTCA_MS10024_S15_R1_001.bw
220517_MS1_CCGTCC_MS10024_S16_R1_001.bw
220517_MS1_CTTGTA_MS10024_S12_R1_001.bw
220517_MS1_GGCTAC_MS10024_S11_R1_001.bw
220517_MS1_GTCCGC_MS10024_S17_R1_001.bw
220517_MS1_GTGAAA_MS10024_S18_R1_001.bw
220517_MS1_TAGCTT_MS10024_S10_R1_001.bw
220517_MS2_AGTCAA_MS10024_S31_R1_001.bw
220517_MS2_AGTTCC_MS10024_S32_R1_001.bw
220517_MS2_ATGTCA_MS10024_S33_R1_001.bw
220517_MS2_CCGTCC_MS10024_S34_R1_001.bw
220517_MS2_CTTGTA_MS10024_S30_R1_001.bw
220517_MS2_GGCTAC_MS10024_S29_R1_001.bw
220517_MS2_GTCCGC_MS10024_S35_R1_001.bw
220517_MS2_GTGAAA_MS10024_S36_R1_001.bw
220517_MS2_TAGCTT_MS10024_S28_R1_001.bw

168_KMT2C_Mut_H3K27me3_broad_a.bw
168_KMT2C_Mut_H3K27me3_broad_c.bw
168_KMT2C_Mut_KDM6A_broad_a.bw
168_KMT2C_Mut_KDM6A_broad_b.bw
168_KMT2C_MUT_P300_a.bw
168_KMT2C_MUT_P300_b.bw
168_KMT2D_Mut_H3K27me3_broad_a.bw
168_KMT2D_Mut_H3K27me3_broad_c.bw
168_KMT2D_Mut_KDM6A_broad_a.bw
168_KMT2D_Mut_KDM6A_broad_b.bw
168_KMT2D_MUT_P300_a.bw
168_KMT2D_MUT_P300_b.bw

168_WT_H3K27me3_broad_a.bw
168_WT_H3K27me3_broad_c.bw
168_WT_KDM6A_broad_a.bw
168_WT_KDM6A_broad_b.bw
168_WT_P300_a.bw
168_WT_P300_b.bw

Genome browser session (e.g. UCSC)

no longer applicable

Methodology

Replicates

For each ChIPseq 2 or 3 biological replicates (defined as 1 passaging (~5 days) between collection of cell line replicates) were sequenced.

Sequencing depth

Libraries were sequenced on an Illumina NovaSeq6000 (Illumina) targeting 40 million 150bp read pairs by the Molecular Biology Core facilities at Dana-Farber Cancer Institute.

Antibodies

H3K4me1 rb 2.5 ug/ChIP abcam ab8895 ChIP
H3K27me3 rb 2.5 ug/ChIP Cell Signaling 9733S ChIP
H3K27ac rb 2.5 ug/ChIP Diagenode C15410196 ChIP
KDM6A rb 2.5 ug/ChIP Cell Signaling 33510S ChIP
P300 rb 2.5 ug/ChIP abcam ab275378 ChIP

Peak calling parameters

All data were analyzed using mm9 genome. Mapping was done using BWA-MEM and peaks were called using macs2 (callpeak --SPMR --broad -B -q 0.01 --keep-dup 1 -g 1.87e9 -f BAMPE --extsize 146 --nomodel). --broad was excluded for H3K27ac peak calling.

Data quality

Minimum number of peaks with >10FC enrichment at 5% FDR was 400 for any dataset. Random genome tracks in each dataset have been visually analyzed for peak shape. Further quality control was performed with ChIPQC. Minimum percentage of reads passing mapping QC filter was 80. No reads were reported as duplicates.

Software

Analysis was performed using the COBRA pipeline (https://bitbucket.org/cfce/cobra/src/master/). For differential peak analysis adj.p.value < 0.05 and log2FC >0.5 were used.novo motifs were considered. For motif heatmap only motifs with -log10 p-value > 25 in at least one sample were plotted. Correlation of differential peaks and gene expression was performed using BETA18. Each individual differential gained or lost peakset was analyzed together with DESeq2 output from RNA-seq of the same cells but different biological replicates using default parameters. For quantification of P300 signal intensities within H3K27ac peaks multiBigwigSummary was used to bin P300 signal of each biological replicate into specific peak regions. Bin count of replicates was averaged and log2 transformed. Density plots for correlation of changed P300 and H3K27ac signal was generated with ggplot. DEseq2 outputs from CoBRA pipeline were used to extract all calculated fold changes within identified peak regions for H3K27ac or P300 in Kmt2c or Kmt2d KO cells compared to WT. Then multiBigwigSummary43 was used with a 100 bin size to calculate fold changes of H3K27ac or P300 in similar region. Quantification of read counts for H3K27me3, P300 and KDM6A within the Mmp3 promoter (TSS +/- 1kb) was performed using featureCounts44 with respective bam files. For comparisons of signal intensities across the Mmp3 cluster locus signal of biological replictaes was first averaged using bigwigAverage43. Then average values were compared using bigwigCompare43 in 1000 bp bin and log2FC mode. Overall signal intensity was then calculated from average values of all bins.

Flow Cytometry

Plots

Confirm that:

☒ The axis labels state the marker and fluorochrome used (e.g. CD4-FITC).

☒ The axis scales are clearly visible. Include numbers along axes only for bottom left plot of group (a 'group' is an analysis of identical markers).

☒ All plots are contour plots with outliers or pseudocolor plots.

☒ A numerical value for number of cells or percentage (with statistics) is provided.

Methodology

Sample preparation

Immediately after collection tissues were smashed with micro pestles and digested for 10 min (brain, bone marrow) or 1 h (tumor, liver and lung) using digestion media (2% w/v collagenase IV, 2% w/v hyaluronidase and 2% w/v BSA in DMEM) at 37 C on a shaker. Solutions were filtered through a mesh, washed with PBS and frozen in 10% DMSO/FBS at -80 C or directly used for flow cytometry. For this, cells were passed through 70 or 100 m cell strainer, incubated with DAPI (1:20,000) and analyzed using a LSR Fortessa (BD Biosciences). Gating strategies can be found in Supplementary Fig 1

Instrument

BD LSRFortessa™ Cell Analyzer

Software

BD FACSDiva was used for data acquisition and FlowJo version 10.9.0 was used for data analysis.

Cell population abundance

No sorting has been performed. Abundance was the primary measurement and thus can be found in the according source data and figures. Purity was controlled with DAPI exclusion.

Gating strategy

Gating strategies can be found in Supplementary Fig 1. In short, cells were selected using FSC-A/SSC-A, single cells were

Gating strategy	selected using SSC-H/SSC-W or FSC-A/FSC-H. DAPI, GFP and mCherry positive or negative cells were selected using unstained negative controls and stained positive controls.

☒ Tick this box to confirm that a figure exemplifying the gating strategy is provided in the Supplementary Information.
