# Supplementary figures and images for: Loss of Kmt2c or Kmt2d drives brain metastasis via KDM6A-dependent upregulation of MMP3
Source: Nat Cell Biol. 2024 Jun 26;26(7):1165–75. doi: 10.1038/s41556-024-01446-3 (PMC11251985; doi:10.1038/s41556-024-01446-3)

Figure 1c

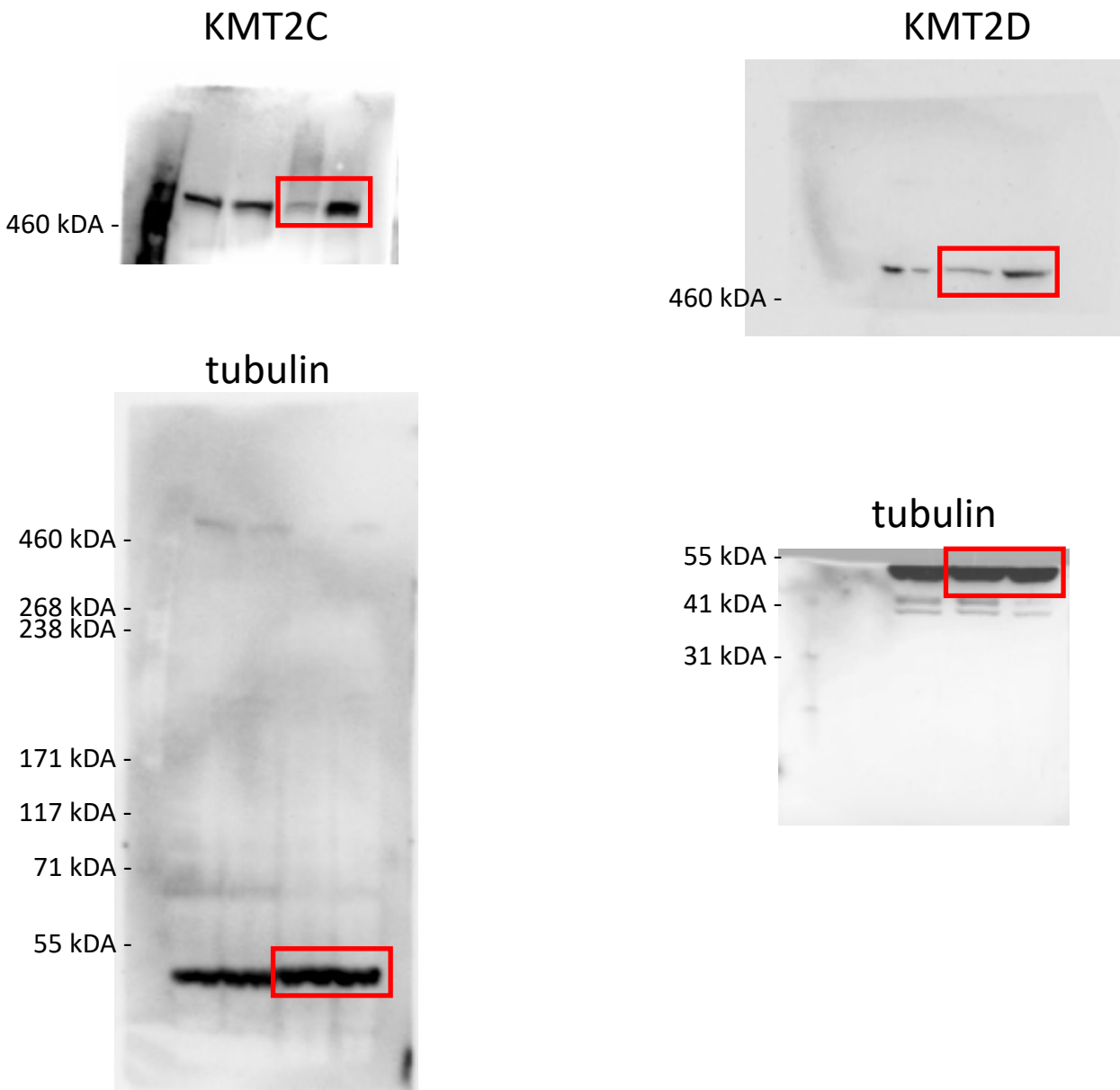

Supplement: Supplementary file 21 — Unprocessed western blots and/or gels. [file 41556_2024_1446_MOESM21_ESM.pdf]

Figure 3c

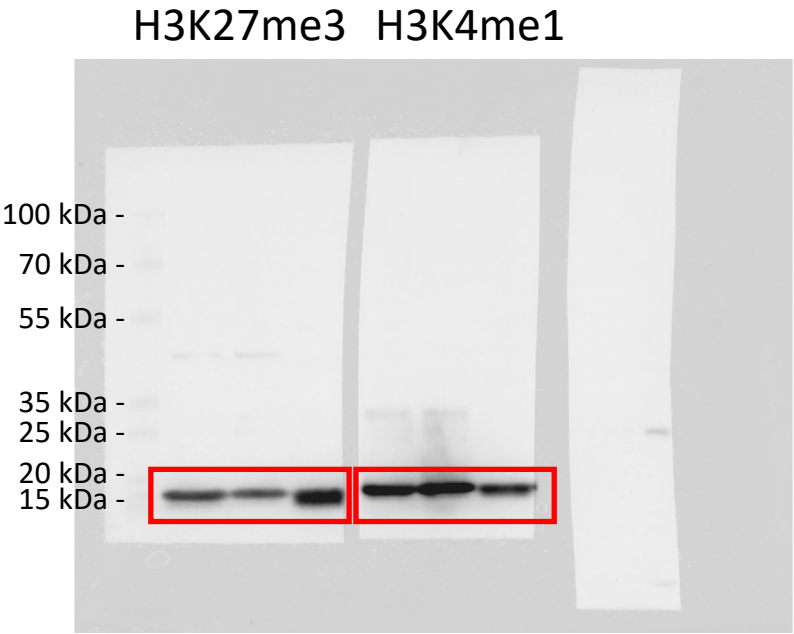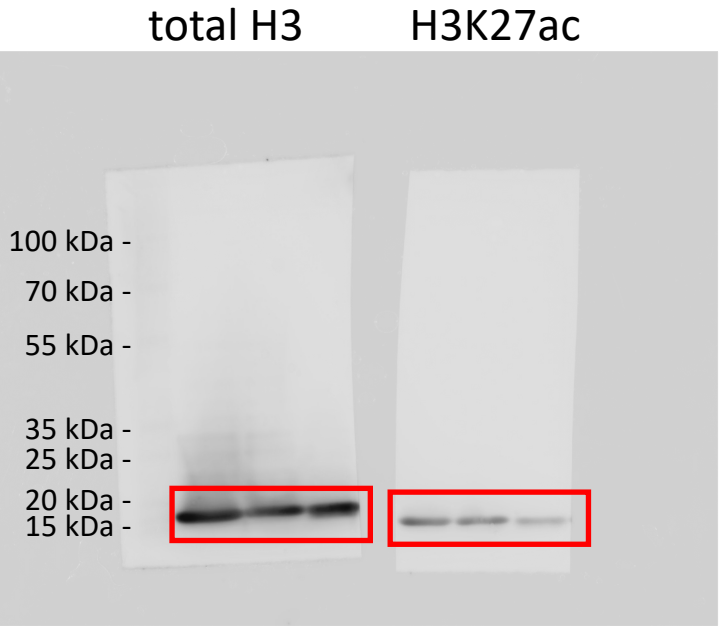

Supplement: Supplementary file 22 — Unprocessed western blots and/or gels. [file 41556_2024_1446_MOESM22_ESM.pdf]

Ext Data Figure 1f

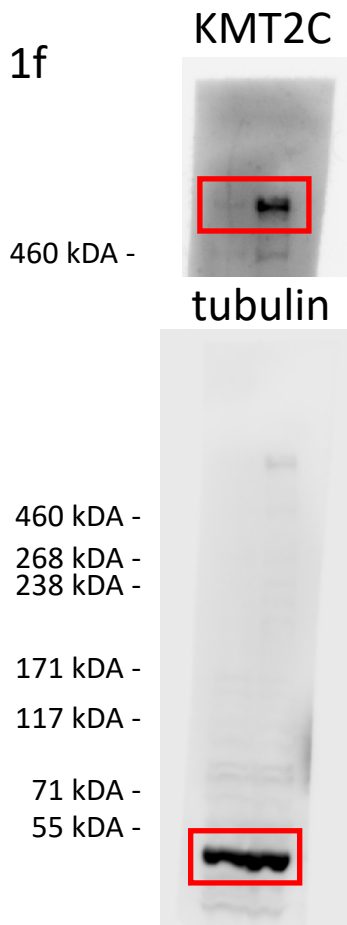

KMT2D

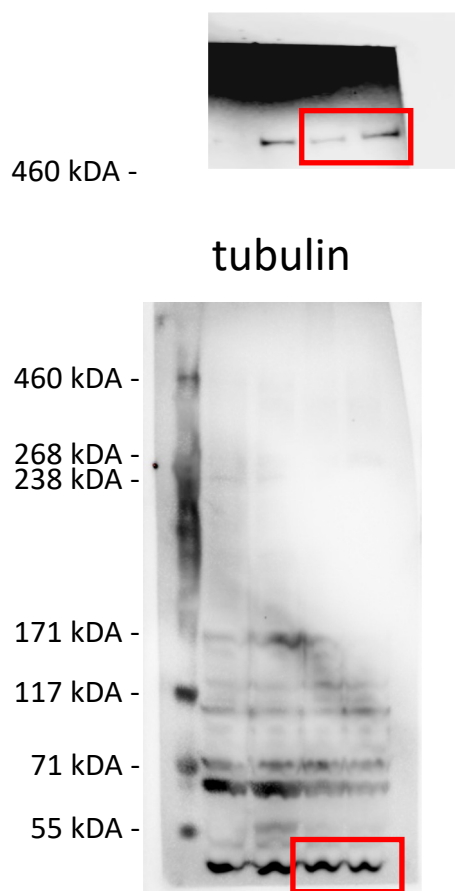

Ext Data Figure 1j

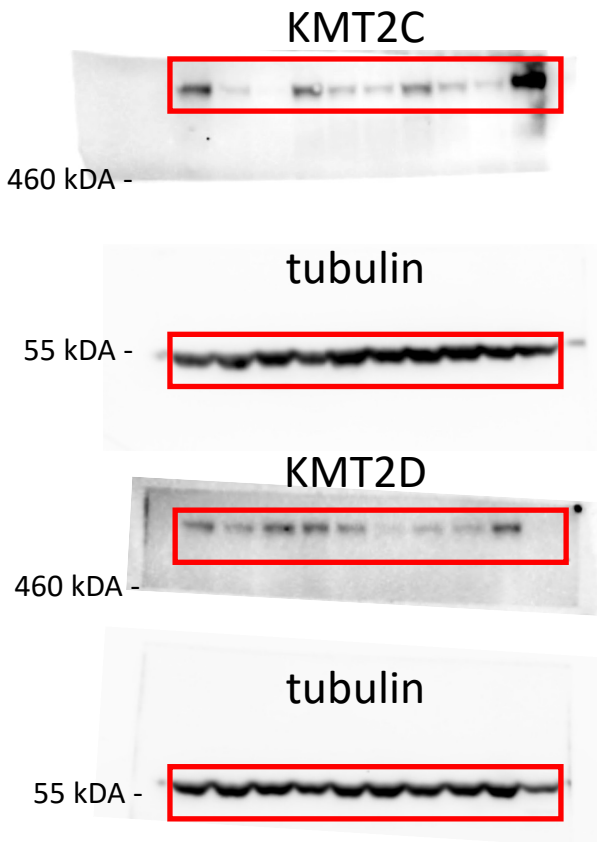

Ext Data Figure 1k

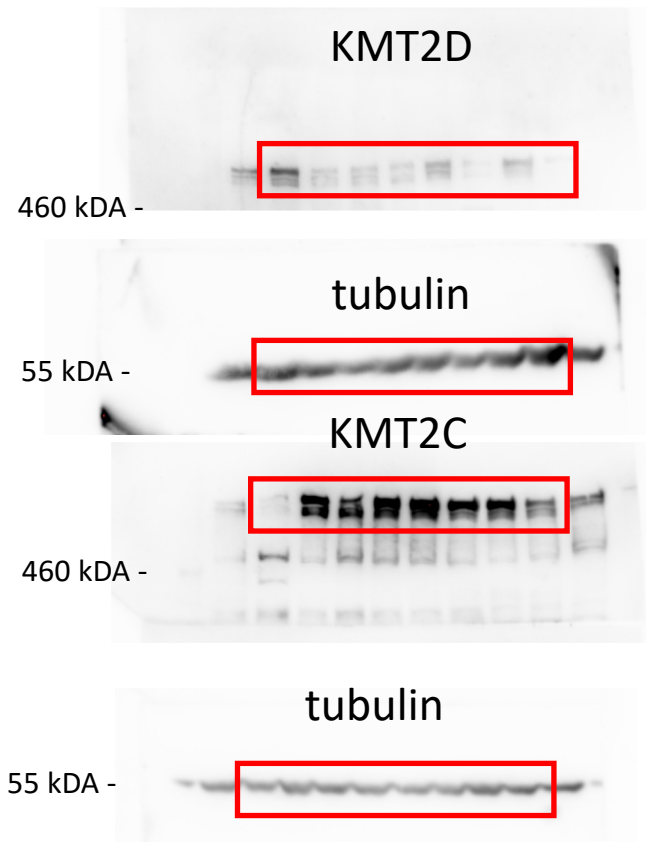

Supplement: Supplementary file 23 — Unprocessed western blots and/or gels. [file 41556_2024_1446_MOESM23_ESM.pdf]

Extended Data Fig 5f

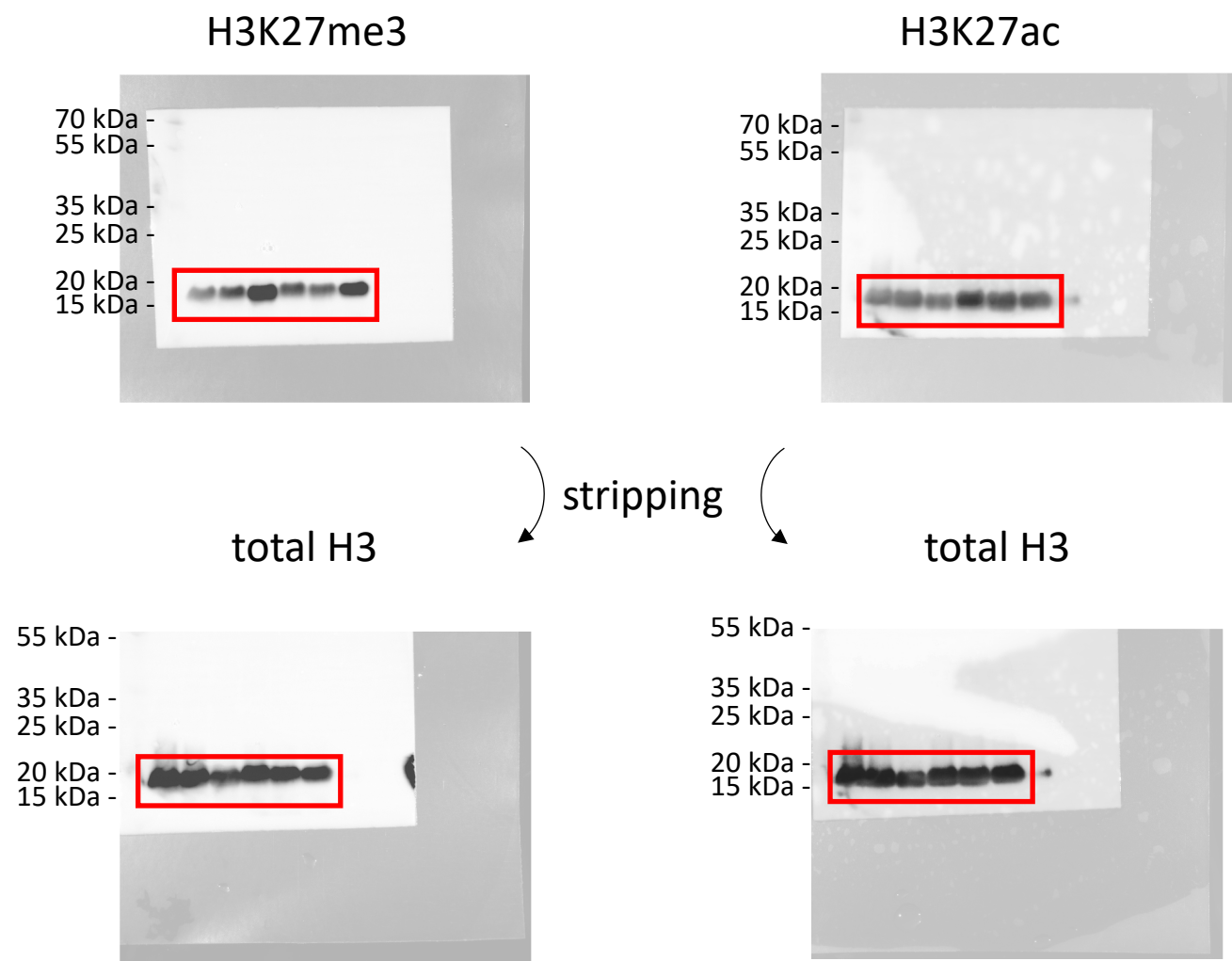

Supplement: Supplementary file 24 — Unprocessed western blots and/or gels. [file 41556_2024_1446_MOESM24_ESM.pdf]

Extended Data Figure 6a

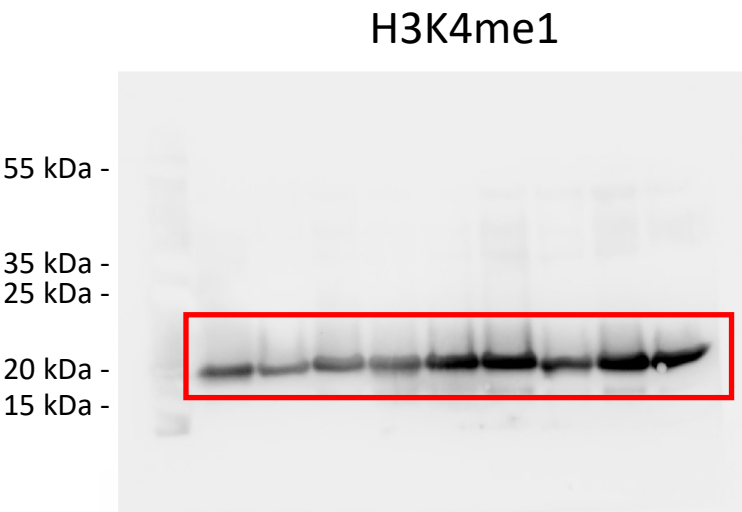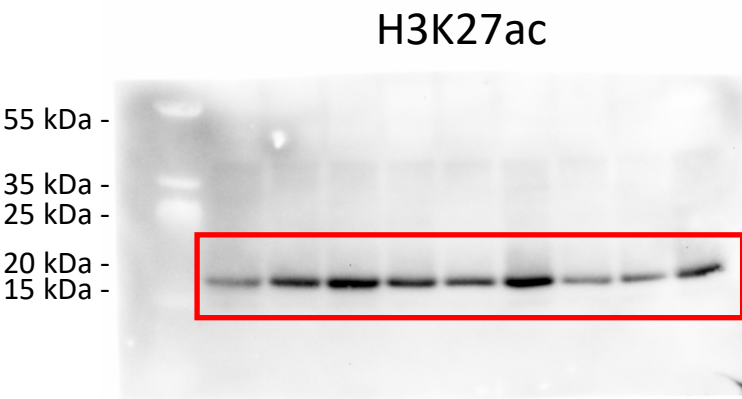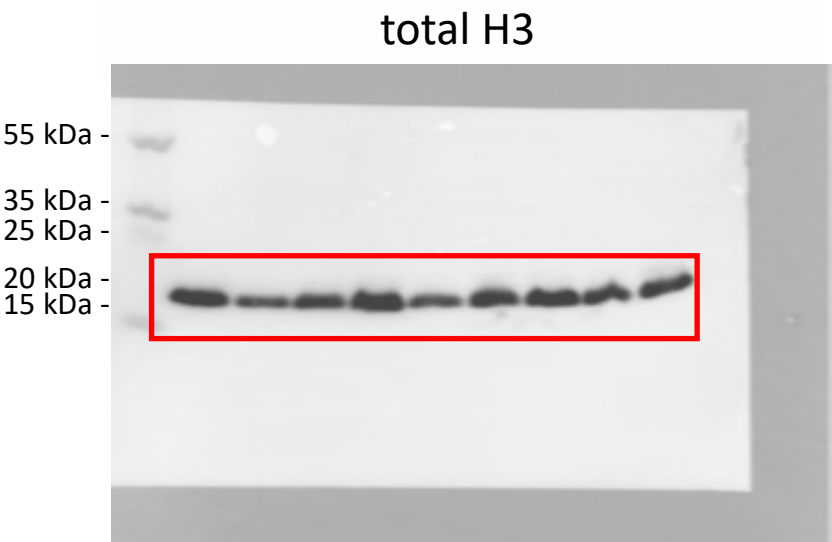

Extended Data Figure 6i

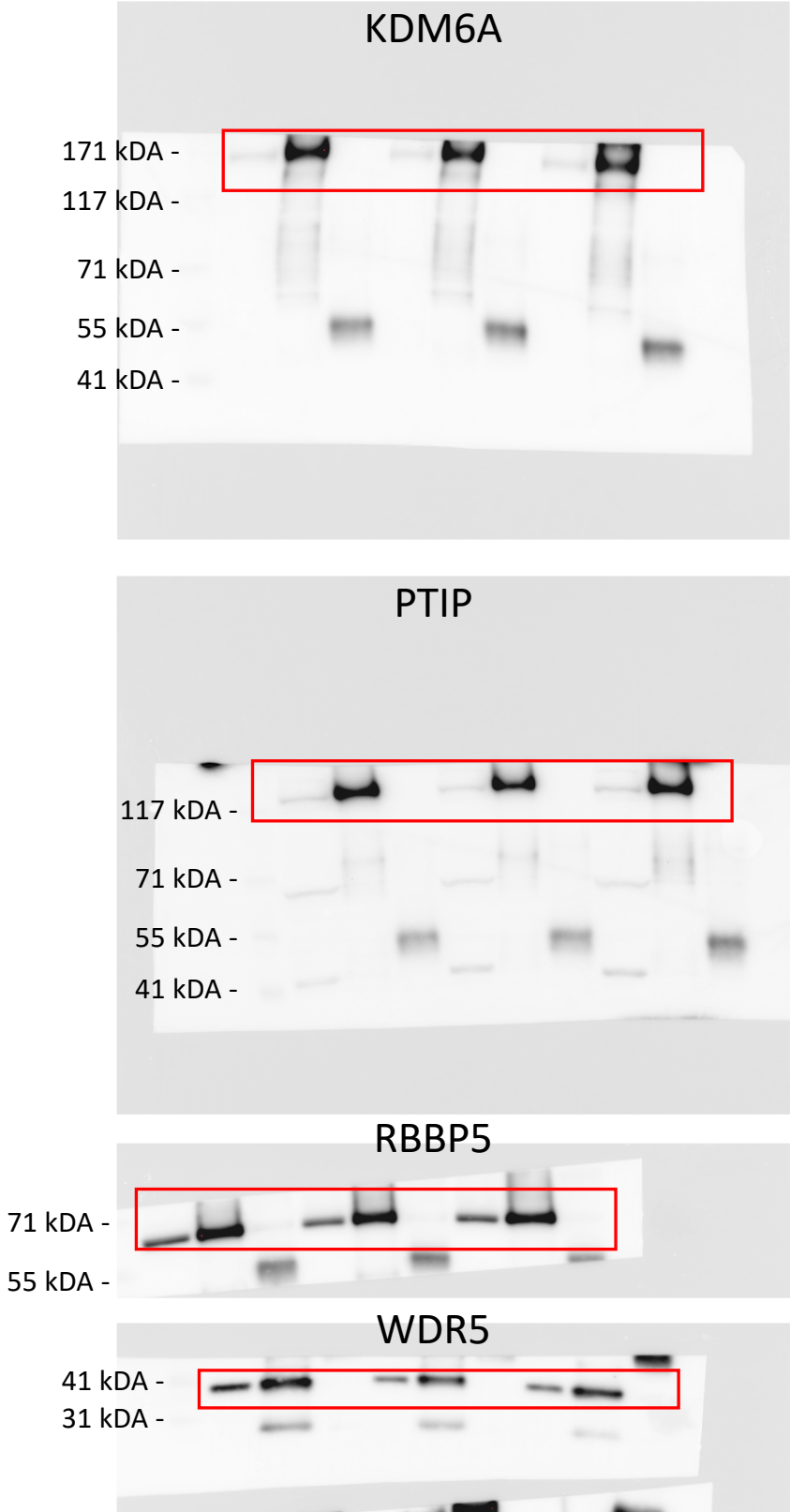

Supplement: Supplementary file 25 — Unprocessed western blots and/or gels. [file 41556_2024_1446_MOESM25_ESM.pdf]
